# Zinc-finger antiviral protein-mediated inhibition of porcine epidemic diarrhea virus growth is antagonized by the coronaviral nucleocapsid protein

**DOI:** 10.3389/fmicb.2022.975632

**Published:** 2022-09-08

**Authors:** Suttipun Sungsuwan, Supasek Kadkanklai, Wuttichai Mhuantong, Anan Jongkaewwattana, Peera Jaru-Ampornpan

**Affiliations:** ^1^Virology and Cell Technology Laboratory, National Center for Genetic Engineering and Biotechnology (BIOTEC), National Science and Technology Development Agency (NSTDA), Khlong Nueng, Pathum Thani, Thailand; ^2^Enzyme Technology Laboratory, National Center for Genetic Engineering and Biotechnology (BIOTEC), National Science and Technology Development Agency (NSTDA), Khlong Nueng, Pathum Thani, Thailand

**Keywords:** coronavirus, virus–host interaction, porcine epidemic diarrhea virus, zinc-finger antiviral protein, nucleocapsid protein

## Abstract

Coronaviruses have long posed a major threat not only to human health but also to agriculture. Outbreaks of an animal coronavirus such as porcine epidemic diarrhea virus (PEDV) can cause up-to-100% mortality in suckling piglets, resulting in devastating effects on the livestock industry. Understanding how the virus evades its host’s defense can help us better manage the infection. Zinc-finger antiviral protein (ZAP) is an important class of host antiviral factors against a variety of viruses, including the human coronavirus. In this study, we have shown that a representative porcine coronavirus, PEDV, can be suppressed by endogenous or porcine-cell-derived ZAP in VeroE6 cells. An uneven distribution pattern of CpG dinucleotides in the viral genome is one of the factors contributing to suppression, as an increase in CpG content in the nucleocapsid (N) gene renders the virus more susceptible to ZAP. Our study revealed that the virus uses its own nucleocapsid protein (pCoV-N) to interact with ZAP and counteract the activity of ZAP. The insights into coronavirus-host interactions shown in this work could be used in the design and development of modern vaccines and antiviral agents for the next pandemic.

## Introduction

Porcine epidemic diarrhea virus (PEDV) is an enveloped virus in the family *Coronaviridae*. The virus infects swine intestinal epithelial cells and causes severe watery diarrhea, vomiting, and dehydration. The high mortality rate due to the infection, especially in suckling piglets, causes severe economic losses in pig production worldwide. Despite continued development, available PEDV vaccines are still suboptimal in their efficacy, making the epidemic a long-term threat to the swine industry. Understanding how a virus antagonizes antiviral factors in a host could provide insights into the innovative design of an effective vaccine as well as a therapeutic intervention for current and emerging outbreaks.

In response to viral infection, hosts have evolved sensing systems that recognize foreign components to trigger innate immune defenses. Zinc-finger antiviral protein (ZAP), also known as Zinc-finger CCCH-type containing antiviral 1 (PARP13 or ZC3HAV1), is a part of the host immune system that acts as a restriction factor against a variety of RNA and DNA viruses (Ficarelli et al., 2021). As products of alternative RNA splicing, the short and long isoforms of ZAP (ZAPS and ZAPL, respectively) share the N-terminal domain, which contains a highly conserved zinc finger domain. The long isoform has an additional C-terminal portion with an inactive poly-(ADP-ribose) polymerase (PARP)-like domain that has been shown to contribute to increased antiviral activity (Schwerk et al., 2019; Kmiec et al., 2021). In general, ZAPS is upregulated as one of the interferon (IFN)-stimulated genes (ISGs) during the type I IFN response, whereas ZAPL is constitutively expressed and less dependent on stimulation by interferon or ISGs (Schwerk et al., 2019; Kmiec et al., 2021).

Zinc-finger antiviral protein selectively binds viral or non-proprietary RNAs with high CpG content, which is abundant in some viruses but underrepresented in the host genome, and recruits cellular endonucleases to degrade the bound viral RNAs (Takata et al., 2017; Ficarelli et al., 2019; Luo et al., 2020). As a result, the amount of viral RNA species decreases significantly, hindering viral protein production and viral replication. To escape detection and subsequent restriction by ZAP, many viruses evolve to suppress CpG dinucleotide content in their own genome (Rima and McFerran, 1997; Cheng et al., 2013). Alternatively, some viruses evolve an effector that counteracts the antiviral function of ZAP. For instance, the NS1 protein of influenza A virus antagonizes the antiviral function of ZAP by preventing ZAP from binding to its viral mRNA target (Tang et al., 2017). Porcine reproductive and respiratory syndrome virus (PRRSV) uses its nsp4 protease to degrade ZAP (Zhao et al., 2020), while enterovirus A71 uses its 3C protease (Xie et al., 2018). Among coronaviruses, only SARS-CoV-2 has been reported to be suppressed by ZAP in a host (Nchioua et al., 2020). However, it remains unclear whether the restriction generally affects all coronaviruses and if the pressure drives them to evolve antagonistic mechanisms against ZAP. The interplay between coronaviruses and ZAP still awaits further investigation.

As a coronavirus, PEDV has a single-stranded positive-sense ∼28-kb RNA genome encoding four structural proteins: spike (S), membrane (M), envelope (E), and nucleocapsid (N). The N protein is one of the most abundant structural proteins produced in infected host cells. Its main function is to oligomerize to form a scaffold associated with viral genomic RNA. This forms the RNA-protein complex that constitutes the inner core of virions. In addition to its main function, N also suppresses host immunity, facilitates viral assembly, and promotes viral genome replication (McBride et al., 2014). We have previously shown that nucleocapsid proteins of porcine alpha-coronaviruses, including PEDV and Transmissible gastroenteritis virus (TGEV), can increase viral RNA content and promote PEDV replication. However, it is not yet clear whether nucleocapsid proteins increase viral RNA production or protect it from degradation.

In this work, the susceptibility of PEDV to ZAP was investigated. We showed that local CpG-rich clusters in the PEDV genome could contribute to suppression by ZAP. We also demonstrated how the virus can use its own nucleocapsid protein to counteract the activity of ZAP. This study altogether demonstrated an alternative function of the nucleocapsid protein in maintaining high viral titers in a host expressing ZAP.

## Materials and methods

### Cell line and plasmid construction

Human embryonic kidney (HEK) 293T (ATCC CRL-3216), African green monkey (VeroE6) cells (ATCC CRL-1586), and their derivatives were grown and maintained in Opti-MEM™ (Gibco™, Thermo Scientific, Waltham, MA, United States) at 37°C and 5% CO_2_. All culture media were supplemented with 10% fetal bovine serum (FBS) and 1% antibiotic-antimycotic solution.

The plasmids for the expression of C-terminal Myc-tagged PEDV-N, TGEV-N, and SADS-CoV-N have been described previously (Jaru-Ampornpan et al., 2017; Sungsuwan et al., 2020). Porcine ZAP (pZAP) genes were PCR-amplified from cDNA reverse transcribed from cellular RNA extracted from porcine alveolar macrophages (PAMs). The forward primer for pZAPL/S (pZAP-F), the reverse primer for pZAPL (pZAPL-R), and the reverse primer for pZAPS (pZAPS-R) are listed in the primer list (Supplementary Table 1). By In-Fusion ligation (Takara Bio, Shiga, Japan), the amplified PCR products were ligated into the pre-digested pCAGGS vector at *Mlu*I and *Kpn*I restriction sites with an indicated C-terminal tag. The virus PEDV-AVCT12-mCherry and their infectious clones (pSMART-BAC- mCherry-PEDVAVCT12 [pPEDV-mCh]) were described previously (Jengarn et al., 2015; Jaru-Ampornpan et al., 2017).

### Generation of zinc-finger antiviral protein-KO cell lines

Zinc-finger antiviral protein-KO HEK293T or VeroE6 ZAP-KO cell pools were generated by the CRISPR-Cas9 approach using a guide RNA (5′-GGCCGGGATCACCCGATCGG-3′). ZAP targeting guide RNA was cloned downstream of the U6 promoter into the lentiCRISPR v2 vector, a gift from Feng Zhang (Addgene plasmid # 52961; RRID:Addgene_52961)^1^ (The resulting plasmid is referred to as lentiCRISPRv2-gZAP-Cas9-P2A-GFP). Wild-type cells were transiently transfected with the lentiCRISPRv2-gZAP-Cas9-P2A-GFP plasmid using FuGENE HD (Promega, Madison, WI, United States), according to the manufacturer’s instructions. At 48 hpt, the transfected cells were sorted based on GFP expression using a fluorescence-activated cell sorter (BD FACSAria™ Fusion, Singapore) to isolate the pool of GFP expressing cells into single isolated cells. Single cell clones that continued to grow were selected for verification of gene knockout. The absence of ZAP expression in the sorted cells was verified by SDS-PAGE/western blot analysis compared to wild-type cells.

### Generation of VeroE6 cell lines stably expressing HA-tagged pZAPL

The ZAP-KO VeroE6 cell line expressing HA-tagged pZAPL was constructed by lentivirus transduction. Lentiviruses carrying the C-terminal HA-tagged pZAPL gene were prepared by co-transfection of pSIN-CSGW-UbEM carrying the inserted pZAPL-HA gene with a packaging plasmid encoding Gag, Pol, Rev, and Tat (pCMV-ΔR8.91) and a plasmid expressing the lentiviral VSV envelope glycoprotein (pMD2.G) into HEK293T cells. At 48 hpt, the supernatants containing the lentiviruses were harvested and filtered through a 0.45-μm filter. The filtered supernatants were then used for transduction of the ZAP-KO VeroE6 cells. A single clone of the transduced cells expressing pZAP was sorted based on GFP expression using a fluorescence-activated cell sorter (BD FACSAria™ Fusion, Singapore) and verified by SDS-PAGE/western blot analysis.

### Viral infection

VeroE6-derived cell lines (5 × 10^5^ cells/ml) plated in a six-well plate were inoculated with 1 ml of the virus at the indicated multiplicity of infection (MOI) for an hour. The inoculum was then removed, and the cells were washed once with PBS and replaced with 2 ml of fresh OptiMEM containing 0.1% TrypLE. The extent of PEDV infection was monitored by mCherry fluorescence under a fluorescence microscope. Cell lysates or supernatants were harvested at the indicated time points for further analysis. To study porcine CoV-N’s ability to rescue ZAP-mediated suppression (Figure 8), VeroE6 cells were transfected with 2 μg of the pCAGGS-plasmid expressing the protein and incubated for 24 h to allow protein expression. Cells were then infected with the virus. To image cytopathic effect (CPE), the infected cells were washed twice with Phosphate-buffered saline (PBS) and fixed with cold 70% acetone and stained with crystal-violet-based plaque staining solution for 20 min. After the staining solution was removed, the plates were washed several times with water and dried before imaging.

### Western blot analysis

Virus-infected or plasmid-transfected cells were harvested and lysed with RIPA lysis buffer (25 mM Tris–HCl pH 7.4, 150 mM NaCl, 1 mM EDTA, 1% NP-40, and 5% glycerol, supplemented with a protease inhibitor cocktail (Halt™ Protease Inhibitor Cocktail, Thermo Fisher Scientific, MA, United States). The mixture was centrifuged at 12,000 g, 4°C for 10 min and the resulting supernatants were mixed with sodium dodecyl sulfate polyacrylamide gel electrophoresis (SDS-PAGE) loading buffer and boiled for 5 min. Proteins from the cell lysates were separated using SDS-PAGE, and then transferred to nitrocellulose membranes (Bio-Rad Laboratories, CA, United States). The membrane was blocked with 5% skim milk prior to incubation with the indicated primary antibodies. Horseradish peroxidase (HRP)-conjugated goat anti-mouse IgG (Biolegend, CA, United States) or (HRP)-conjugated donkey anti-rabbit IgG (Biolegend, CA, United States) were used as secondary antibodies. Primary antibodies used in this study included mouse-anti-Myc (Thermo Fisher Scientific, MA, United States), rabbit-anti-FLAG (ab1162, abcam, Cambridge, United Kingdom), rabbit-anti-HA (ab9110, abcam, Cambridge, United Kingdom), and mouse-anti-PEDV-N (SD 6–29, Medgene labs, SD, United States), rabbit polyclonal anti-Zinc finger antiviral protein (ab154680, abcam, Cambridge, United kingdom).

### Virus titration (TCID_50_ assay)

VeroE6 cells stably expressing TGEV-N-FLAG (Sungsuwan et al., 2020) were plated overnight in 96-well plates to obtain monolayers of the cells. The culture media were removed, and the cells were washed once with PBS. Supernatants obtained from infected cells were prepared at 10-fold serial dilutions in OptiMEM containing 0.1% TrypLE. The diluted virus was added to the pre-seeded cells (eight wells for each dilution). At 72 h post-infection (hpi), the infected cells were examined by mCherry expression under a fluorescence microscope. The TCID_50_ of the viral titers was determined using the Reed-Muench method (Reed and Muench, 1938).

### Generation of sequences encoding the porcine epidemic diarrhea virus-N gene with varied CpG content

To generate PEDV-N sequences with high CpG content, an in-house algorithm was developed based on the concept of codon pair bias deoptimization introduced by Coleman et al. (2008) via python3,^2^ to recode a gene by replacing native codon pairs with synonymous codon pairs and obtain a sequence with an expected codon-paired bias (CPB) value. The generated codon-paired bias deoptimized (CPD) sequences were further analyzed using SSE software (Simmonds, 2012) and sorted according to the difference in RNA folding energy compared to the original sequence. Selected sequences with the highest similarity in RNA folding energy profile compared to that of the native N gene sequence were finally selected. The selected sequences were chemically synthesized *de novo* by an oligonucleotide synthesis service provider (Integrated DNA Technologies, Inc.; IDT, Singapore), and used for subsequent experiments.

### Construction of infectious clones with CPD-N genes and rescues of reverse genetics-derived CPD-porcine epidemic diarrhea virus

Oligonucleotide fragments extending from the early S gene to the 3′ end of the M gene and fused to the full length of various CPD-N genes were obtained by overlap PCR extension of the S-mCherry-E-M fragments and the corresponding full length CPD-N (Figure 3D). The purified PCR product was inserted by In-Fusion ligation into a pre-digested vector of pPEDV.mCh.*Mlu*I.3UTR at *Pac*I and *Mlu*I restriction sites. To rescue the virus from the infectious clone, HEK293T cells were transfected with 3 μg of the infectious clone plasmid carrying the indicated CPD-N sequence and 0.5 μg of PEDV N expressing plasmid in order to enhance virus production (Liwnaree et al., 2019). FuGENE^®^ HD (Promega, Madison, WI, United States) was used as a transfection reagent, according to the manufacturer’s instructions. At 72 hpt, supernatants were transferred to adsorb onto VeroE6-PEDV N cells for 1 h at 37°C. The inocula were then removed, and the cells were washed twice with PBS. 2 ml OptiMEM containing 0.1% TrypLE (Thermo Fisher Scientific, MA, United States) was added, and the cells were kept in the incubator at 37°C and 5% CO_2_ for 72 h. The rescued viruses were harvested by the freeze-thaw method and titrated by the TCID_50_ method. The harvested viruses were kept in −80°C until use.

### Co-immunoprecipitation

HEK293T cells in 6-well plates were transfected with 0.5–1.0 μg of plasmids using FuGENE HD (Promega, Madison, WI, United States), according to the manufacturer’s instructions. The cells were lysed on ice in lysis buffer (50 mM Tris–HCl pH 8.0, 150 mM NaCl, 5 mM EDTA, 1% NP-40 supplemented with a protease inhibitor cocktail). The lysates or mixed lysates were centrifuged at 20,000 g for 10 min at 25°C. The lysates were aliquoted into 50 μl for immunoblot and 150 μl for co-immunoprecipitation. Co-immunoprecipitated lysates were incubated with 25 μg of agarose beads conjugated to the indicated antibody (Pierce™ anti-c-Myc or HA-Epitope Tag Antibody agarose, Thermo Fisher Scientific, IL, United States) overnight at 4°C under rotation. The lysates were washed three times with wash buffer (50 mM Tris–HCl pH 8.0, 550 mM NaCl, 5 mM EDTA, 1% NP-40), and then eluted with 25 μl 2X non-reducing dye then boiled for 5 min and supplemented with 5 μL 0.5M DTT. Proteins were analyzed by SDS-PAGE/western blot analysis.

### Immuno-fluorescence imaging

VeroE6 ZAP-KO cells plated on glass coverslips were transfected with the indicated plasmids. At 48 hpt, cells were fixed with 4% paraformaldehyde at 4°C for 20 min and washed three times with PBS. The fixed cells were permeabilized and blocked for 1 h with 1% BSA, 1% FBS, and 0.1% Triton-X in PBS. Then, cells were stained with rabbit anti-HA (pZAPL) (1:500) and mouse anti-PEDV-N (1:500) diluted in 1% BSA in PBS for 1 h. After washing three times with PBS, cells were stained with AlexaFluor-488 goat anti-mouse IgG antibody (1:500) or AlexaFluor-568 goat anti-rabbit IgG antibody (Invitrogen, Thermo Fisher Scientific, OR, United States) (1:500) diluted in 1% BSA in PBS for 1 h and then washed with PBS three times. Glass coverslips were mounted on slides using ProLong™ Diamond Antifade Mountant with DAPI (Invitrogen, Thermo Fisher Scientific, OR, United States). Protein localization was observed and analyzed using Olympus Fluoview-1000 confocal microscopy.

### Luciferase assays measuring zinc-finger antiviral protein activity

Synthetic oligonucleotides of each ZAP-responsive sequence (ZRS) were inserted into the pGL3-SV40-Luc (Promega, Madison, WI, United States) plasmid as a 3′UTR downstream of the Luciferase encoding gene as described previously (Guo et al., 2004a; Chen et al., 2012). A ZAP-sensitive sequence derived from Sindbid virus, Na, was used as a positive control (Guo et al., 2004a). For the experiment to determine ZAP activity in the presence of the various pCoV-N, a reporter plasmid carrying N-0.10 as ZRS was used as it showed the highest ZAP sensitivity in our study. Each construct was co-transfected with plasmids carrying pZAPL or pZAPS and the empty vector- or pCoV-N expressing plasmid as indicated into ZAP-KO HEK293T cells. A plasmid expressing Renilla luciferase, pRL-TK, was used to normalize transfection efficiency. At 48 hpt, cells were lysed, and luciferase activity was measured using the Dual-Luciferase Reporter Assay System (Promega, Madison, WI, United States) according to the manufacturer’s instructions with an EnVision plate reader (PerkinElmer, MA, United States). Fold inhibition was calculated as the normalized luciferase activity in ZAP-KO HEK293T cells divided by the normalized luciferase activity in ZAP-expressing cells.

### Statistical analysis

All data with statistical analysis were analyzed with GraphPad Prism 7.0 (GraphPad Software Inc., La Jolla, CA, United States). All results were presented as means ± standard errors of the means (SEM); *p* values of <0.05 were considered statistically significant.

## Results

### Porcine epidemic diarrhea virus replication was suppressed by zinc-finger antiviral protein in VeroE6 cells

**FIGURE 1:**
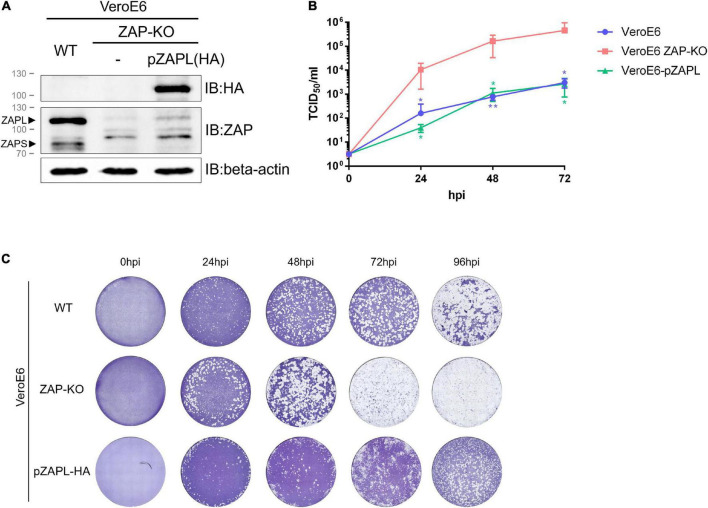
Replication of porcine epidemic diarrhea virus (PEDV) is suppressed in VeroE6 cells by an endogenous or porcine cell-derived long isoform of zinc-finger antiviral protein (ZAP). **(A)** The VeroE6 ZAP-KO cell line was verified by western blot analysis. To generate a stable VeroE6 cell expressing only the HA-tagged long isoform of pZAP (VeroE6-pZAPL), VeroE6 ZAP-KO cells were transduced using the lentiviral transduction method. SDS-PAGE/western against the HA tag confirmed expression of HA-tagged pZAPL in the transduced cells. **(B)** VeroE6, VeroE6 ZAP-KO, or VeroE6-pZAPL cells were infected with PEDV (AVCT12 strain) at MOI = 0.0001. PEDV growth kinetics were monitored at the indicated time points using the TCID_50_ assay. Values shown are averages ± SEM of three independent experiments (Student’s *t*-test, **p < 0.05, **p < 0.005). **(C)** Representative images showing the spread of virus at different time points post-infection were visualized with cytopathic effect (CPE) by crystal violet staining.

We first determined whether PEDV was sensitive to ZAP. The ZAP gene (ZC3HAV1) was knocked out in VeroE6 cells by a CRISPR-Cas9-based approach. The selected knockout clone of the cell line (designated VeroE6 ZAP-KO) was confirmed by western blot and ZAP-encoding RNA expression (Figure 1A and Supplementary Figure 1). Because PEDV normally infects pigs, we also wanted to determine the degree of inhibition by a more biologically relevant porcine host factor. A porcine-derived ZAP gene (pZAP) was cloned from porcine alveolar macrophage cells (PAMs) and transduced into VeroE6 ZAP-KO cells to generate a stable VeroE6 cell line expressing only the HA-tagged long isoform of pZAP (designated as VeroE6-pZAPL). Since pZAPL cannot be recognized by anti-human-ZAP antibodies, expression of HA-tagged pZAPL in the transduced VeroE6 cells was confirmed by SDS-PAGE/western blot analysis against the HA tag (Figure 1A). PEDV was inoculated into wild-type VeroE6, VeroE6 ZAP-KO, or VeroE6-pZAPL cells at MOI = 0.0001. The growth rate of PEDV determined by TCID_50_ (Figure 1B) and viral spread at different time points by plaque imaging (Figure 1C) showed that the virus replicated significantly faster in VeroE6 ZAP-KO cells than in the wild-type or VeroE6-pZAPL cells. These results suggest that PEDV can be suppressed by ZAP derived from either the African green monkey host (VeroE6) or a porcine host (PAMs).

### The porcine epidemic diarrhea virus genome contains an uneven distribution pattern of CpG dinucleotides similar to other human coronaviruses

**FIGURE 2:**
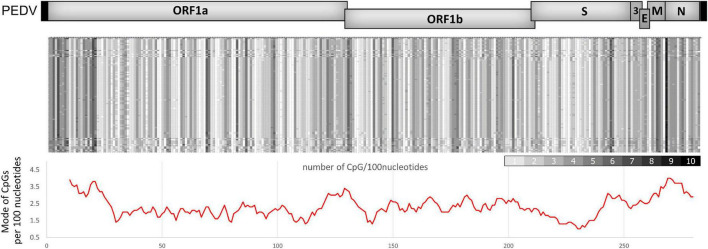
CpG dinucleotide distribution in porcine epidemic diarrhea virus (PEDV) genomes. **(top)** Representative complete genome sequences of various PEDV strains were aligned (NCBI accession numbers are listed in the Supplementary material). The number of CpGs per 100 nucleotides of each segment was analyzed. Results were shown in a heat map (white = 0 CpGs/100-bp, black = 10 CpGs/100-bp). **(Bottom)** Mode of CpGs per 100-bp from the aligned sequences in each region of the PEDV genomes. The graph below the heat map was plotted by a moving average trend line for every 1,000-bp (10 values of CpGs/100-bp) showing high CpG regions in the early 5′ region of ORF1a, the frameshift region between ORF1a and ORF1b, and the structural genes downstream of the spike gene.

Coronaviruses generally contain low levels of CpG, considering the average of their genomes (Xia, 2020). However, SARS-CoV-2 replication has been reported to be suppressed by endogenous ZAP (Nchioua et al., 2020). Therefore, we hypothesized that the suppression of coronaviruses, including PEDV, might be due to an uneven distribution of CpG clusters in the viral genome. We analyzed the CpG distribution in the genomes of various PEDV strains. The analysis shows that there are common areas where a high density of CpG content is clustered. These areas include the early 5′region of ORF1a, the frameshift region between ORF1a and ORF1b, and the structural genes downstream of the spike gene, particularly in the N gene (Figure 2). The result shows a similar pattern of distribution of CpG content in their genomes as in SARS-CoV-2 (Nchioua et al., 2020; Zimmer et al., 2021). This led us to speculate that these highly localized CpG contents could be targeted by ZAP.

**FIGURE 3:**
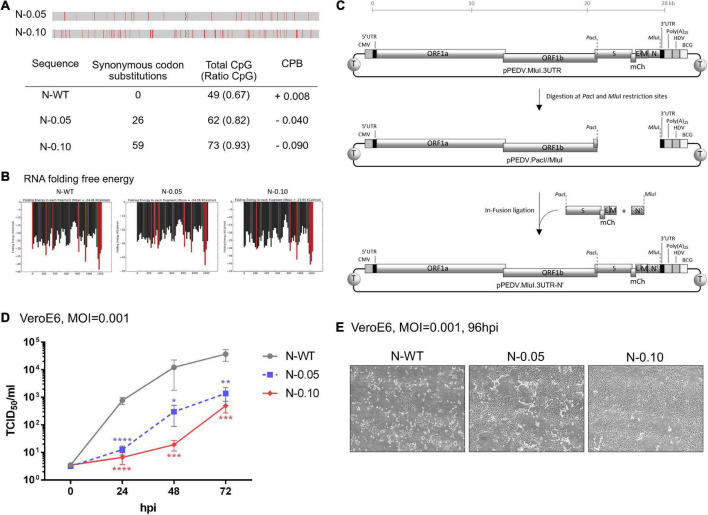
Increasing CpG content in the N gene results in slower growth of porcine epidemic diarrhea virus (PEDV) in VeroE6 cells. **(A)** Analysis of generated nucleotide sequences encoding PEDV-N protein with varied CpG dinucleotides. Red lines in a sequence bar display silent mutations where the original nucleotides are replaced to increase CpG content. Synonymous codon substitutions, CpG dinucleotide compositions, and codon-paired bias (CPB) values of each sequence are listed in the Table. **(B)** RNA folding free energy profiles for each 100- and 20-base window of the generated CPD-N sequences compared to the native N gene sequence. The red bars indicate the high (<–30 Kcal/mol) free energy of RNA folding. **(C)** Construction of infectious clones using the N genes with high CpG content. PCR-amplified products of an oligonucleotide fragment from the S gene to the M gene (S-mCherry-E-M) and another fragment of various N genes (N′) were inserted into a pre-cut vector containing the rest of the viral genes (pPEDV.mCh.*Mlu*I.3UTR) by the In-Fusion ligation method. **(D)** VeroE6 cells were infected with each recombinant PEDV (MOI = 0.001) and virus replication was monitored at the indicated time points by the TCID_50_ assay. Values shown are averages ± SEM of three independent experiments. **(E)** Representative images of virus spread in the infected cells at 96 hpi are shown.

### Modulation of CpG content in the N gene affects growth rates of recombinant porcine epidemic diarrhea virus in wild-type host cells

Based on the above analysis, we hypothesized that altering the CpG cluster in the viral genome would affect viral growth in wild-type cells in a ZAP-dependent manner. For instance, increasing CpG clusters would further attenuate the mutant virus. We chose to increase CpG content in the N gene because it could affect viral transcriptional regulatory sequences in other suspected areas of high CpG content, such as the 5′UTR or ribosomal frameshift region (TRS). Nucleotide sequences encoding the viral N gene with increased CpG content without changing its amino acid sequence were generated with controlled RNA folding energy to avoid the effects of RNA folding on protein expression (Figures 3A,B). Two sequences were selected, as shown in Figure 3A. While the native sequence contains 49 CpG (67%) with a codon-paired bias (CPB) value (Moura et al., 2007) = +0.008, the generated sequences, N-0.05 and N-0.10, contain 62 CpG (82%, CPB = −0.04) and 73 CpG (93%, CPB = −0.09), respectively.

To determine how increasing CpG density in the N gene affects viral growth in wild-type cells, we generated recombinant PEDVs carrying these synthetic sequences and measured their replication kinetics. The oligonucleotides carrying the generated sequences were cloned into the PEDV infectious clone according to the method shown in Figure 3C. The infectious clone plasmids were transfected into HEK293T cells to generate infectious particles. The recombinant viruses were passaged and verified by RT-PCR sequencing of viral RNA (data not shown). We inoculated the resulting viruses at MOI = 0.001 onto VeroE6 cells to determine the replication rate of each virus by TCID_50_ assay. The generated viruses grew at varied rates that were inversely correlated with the level of CpG content in the N gene (Figures 3D,E). The results in this section suggest that increasing the CpG content in the N gene of PEDV can suppress viral growth in a susceptible cell line.

### Growth of porcine epidemic diarrhea virus carrying high-CpG content in the N gene is suppressed by porcine zinc-finger antiviral protein

**FIGURE 4:**
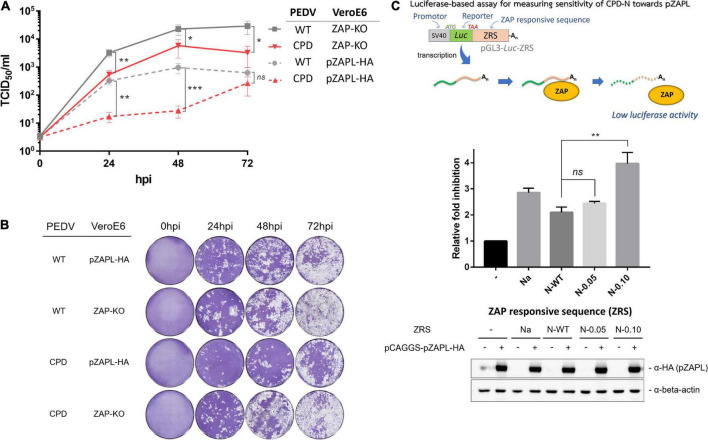
The growth of CPD-porcine epidemic diarrhea virus (PEDV) is suppressed by porcine zinc-finger antiviral protein (ZAP). **(A)** VeroE6 ZAP-KO or VeroE6-pZAPL cells were infected with CPD-PEDV at MOI = 0.001. Viral growth kinetics in VeroE6 ZAP-KO or VeroE6-pZAPL were monitored at the indicated time points using the TCID_50_ assay. Values shown are averages ± SEM of three independent experiments (Student’s *t*-test, **p < 0.05, **p < 0.005, ***p < 0.0005). **(B)** Representative viral spread at varied time points from A) was shown as images of CPE. **(C) (Top)** Schematic representation of the luciferase-based assay used to measure the sensitivity of each ZRS to pZAPL. **(Middle)** Each reporter plasmid containing ZRS was transfected into the ZAP-KO-293T cells along with a transfection normalizing plasmid, pRL-TK, and an empty control plasmid or a pZAPL-expressing plasmid. At 48 hpt, the transfected cells were lysed and luciferase activity was measured using the dual-luciferase reporter assay. The sensitivity of each ZRS to pZAPL was compared as the inhibition fold, which was calculated as described in methods. Inhibition data shown are the means ± SD of three independent measurements (Student’s *t*-test, **p < 0.005). **(Bottom)** SDS-PAGE/western blot showing expression of pZAPL in each condition.

Based on the above results, PEDV with N-0.10 (designated as CPD-PEDV) was selected as it grew the slowest in WT-VeroE6 to further investigate the sensitivity of increased CpG content in the N gene on viral growth against the corresponding porcine ZAP. To test whether the suppressed growth of CPD-PEDV is caused by ZAP in the host, we compared the growth of CPD-PEDV and WT-PEDV in cells lacking or expressing ZAP. CPD-PEDV or WT-PEDV (MOI = 0.001) were inoculated onto either VeroE6 ZAP-KO or VeroE6-pZAPL. Supernatants of infected cells were collected to determine the virus titers at varied time points by TCID_50_ (Figure 4A), and the spread of viruses was shown by images of the CPE (Figure 4B). The results show that, while WT-PEDV was suppressed by pZAPL by 1–1.5 logs (Figure 4A, gray lines), CPD-PEDV is even more sensitive to pZAPL, resulting in 1.5–2.5 log decrease in titers (Figure 4A, red lines). In ZAP-KO cells, only a small viral growth difference (∼0.5–1.0 logs) is observed between the two viruses (Figure 4A, solid lines), possibly due to ZAP-independent effects (e.g., different translation efficiency or RNA stability). However, in cells expressing pZAPL, the growth difference is significantly greater, especially at early time points (∼1.5 logs) (Figure 4A, dashed lines), indicating a ZAP-dependent effect on the CPD-virus at early time points in the viral replication process. These results suggest that the reduced replication of PEDV with a high CpG content in the N gene is affected by ZAP-mediated suppression.

To provide additional evidence that the selected sequences in CPD-PEDV are targeted according to the increased CpG content of ZAP, we applied a luciferase-based assay previously used to determine the sensitivity of RNA sequences to ZAP (Guo et al., 2004a; Chen et al., 2012). The assay is based on a reporter plasmid encoding a luciferase gene fused to an untranslated region containing a ZAP-responsive sequence (ZRS) (Figure 4C). In this assay, ZAP targets the ZRS and subsequently degrades total RNA, resulting in inhibition of luciferase signaling. The more ZAP-sensitive the sequences are, the stronger the luciferase inhibition will be. The synthesized N gene with varied CpGs was cloned into a non-translated region downstream of the luciferase gene in the pGL3-Luc plasmid as was done previously (Guo et al., 2004a; Chen et al., 2012; Figure 4C). The resulting Luciferase-based reporter plasmids (pGL3-Luc-ZRS) with various CpG-containing sequences were transfected into ZAP-KO HEK293T cells with a transfection normalization plasmid expressing Renilla luciferase (pRL-TK) and an empty pCAGGS vector or a pCAGGS plasmid expressing HA-tagged pZAPL (pCAGGS-pZAPL-HA). A previously reported ZAP-sensitive sequence, Na, was used as a positive control (Guo et al., 2004a). At 48 hpt, the cells were lysed, and luciferase activities were measured by the Dual-Luciferase Reporter Assay. The ZAP sensitivity of each generated sequence is presented as the inhibition fold as done previously in other studies (Guo et al., 2004b; Chen et al., 2012). Intermediate increase in CpG dinucleotides in N-0.05 slightly increases ZAP sensitivity, while the reporter plasmid with the highest CpGs, N-0.10, highly enhances the sensitivity toward pZAPL (Figure 4C). This result supports the hypothesis that the increased CpG dinucleotides in the N gene of CPD-PEDV are correlated with the higher ZAP-sensitivity of the virus.

### Porcine epidemic diarrhea virus nucleocapsid protein interacts with pZAPL

From the above results, we observed delayed replication of the CPD-virus in a host expressing pZAPL at an early time point (24–48 hpi) (Figures 4A,B) compared with the ZAP-KO cells. However, the virus accelerated its growth at the late stage of replication (72 hpi). We next asked whether PEDV could use its own viral factors, which accumulate during prolonged replication, to counteract the suppression exerted by ZAP.

Among viral structural proteins, nucleocapsid protein (N) is the most likely candidate, as an interactome study by Zheng et al. (2021) showed that N of SARS-CoV-2 can interact with ZAP. Moreover, several studies have shown that N from human coronaviruses can interact with TRIM25, an essential co-factor to promote the function of ZAP (Hu et al., 2017; Li et al., 2017; Chang et al., 2020). We now aimed to determine whether PEDV N has an antagonistic function against ZAP.

**FIGURE 5:**
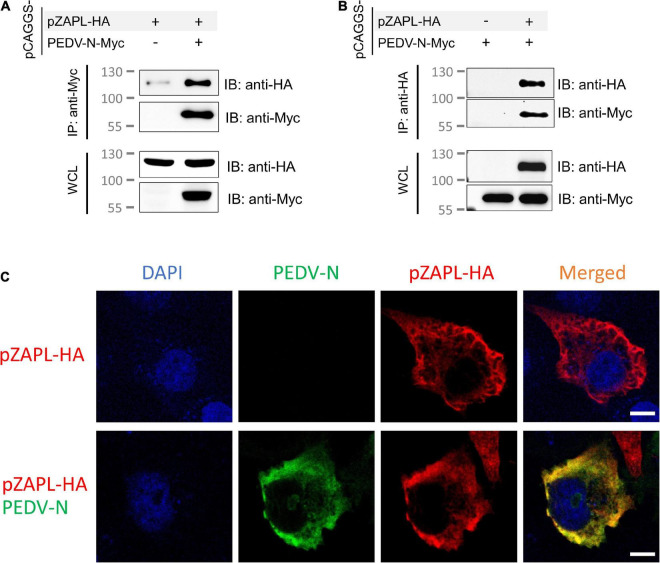
pZAPL interacts with PEDV-N. **(A,B)** Cell lysates from ZAP-KO HEK293T transfected with pCAGGS-pZAPL-HA or pCAGGS-PEDV-N-Myc were mixed as indicated. A co-IP experiment was performed with agarose beads conjugated with anti-Myc- **(A)** or anti-HA **(B)** antibodies. Prey proteins were analyzed by western blot with the indicated antibodies. **(C)** Co-localization of PEDV-N with pZAPL. VeroE6 cells were co-transfected with pCAGGS-PEDV-N and pCAGGS-pZAPL-HA. Confocal microscopy images of the transfected cells were taken at 48 hpt to examine the localization of the indicated proteins (scale bar = 10 μm).

First, we examined the interaction between PEDV-N and pZAPL. A co-IP experiment was performed to determine whether they interact with each other. Cell lysates from ZAP-KO HEK293T transfected with pCAGGS-pZAPL-HA or pCAGGS-PEDV-N-Myc were mixed and incubated. By pulling PEDV-N-Myc in the co-IP setup, we found that PEDV-N can pull pZAPL-HA (Figure 5A) and vice versa when pZAPL-HA is used as bait (Figure 5B). The co-IP result indicated that PEDV-N can be associated with pZAPL. Co-localization between these proteins in cells was demonstrated by confocal imaging (Figure 5C).

### Nucleocapsid proteins of porcine alpha-coronaviruses suppress the function of porcine cell-derived zinc-finger antiviral protein

Next, we investigated how nucleocapsid proteins from porcine coronaviruses affect the function of ZAP. In this experiment, we used a luciferase-based assay using the reporter plasmid described above [pGL3-Luc-ZRS(N-0.10)] (Figure 4C). To test how different ZAP isoforms behave, short and long isoforms of ZAP, derived from porcine cells, were cloned from porcine alveolar macrophage cells (PAMs). Both isoforms showed ZAP activity in this assay setup, with pZAPL exerting stronger inhibition than pZAPS (Supplementary Figure 2).

**FIGURE 6:**
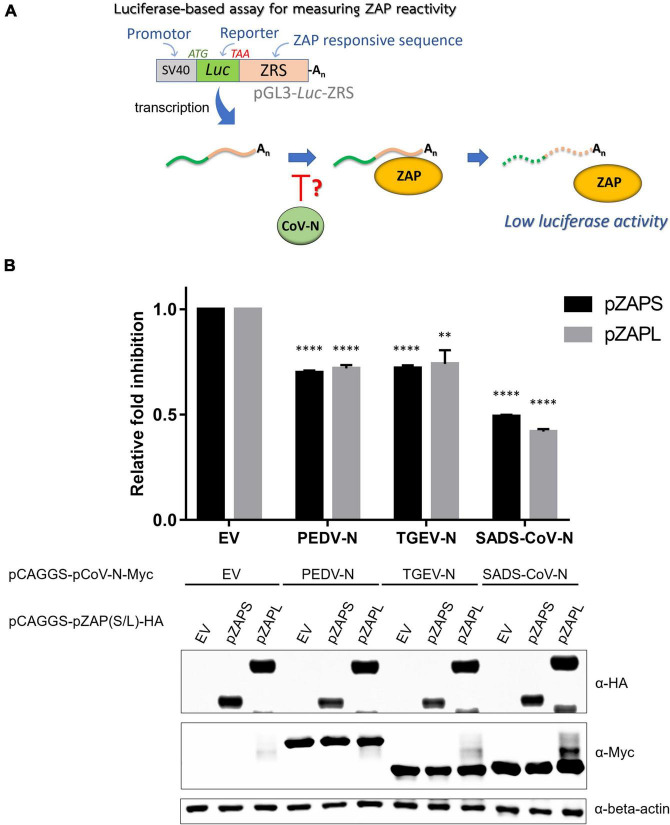
Nucleocapsid proteins of porcine alpha-coronaviruses suppress the function of porcine cell-derived zinc-finger antiviral protein (ZAP). **(A)** Schematic representation of the procedure of a luciferase-based assay to measure the activities of pZAPL and -S in the presence of the various pCoV-N. **(B)** To assess the ability of pCoV-N to antagonize ZAP activity, pGL3-Luc-ZRS was co-transfected with pZAPS or -L and the indicated pCoV-N expressing plasmids. A plasmid expressing Renilla luciferase, pRL-TK, was used to normalize transfection efficiency, and an empty pCAGGS vector (EV) was used as a control plasmid. At 48 hpt, cells were lysed, and luciferase activity was measured. Relative fold inhibition is fold inhibition (described in “Materials and methods”) in the presence of the respective pCoV-N divided by fold inhibition of cells with EV. Data shown are the means ± SD of three independent measurements (Student’s *t*-test, **p < 0.005, ****p < 0.00005). **(Bottom)** SDS-PAGE/western blot showing expression of pZAPL in each condition.

To investigate the effect of pCoV-N on the activity of ZAP, ZAP-KO HEK293T cells were co-transfected with the reporter plasmid [pGL3-luc-ZRS(N-0.10)], the pCAGGS plasmid expressing HA-tagged pZAPL (pCAGGS-pZAPL-HA) and the plasmids expressing various pCoV-N in the presence of the plasmid expressing Renilla luciferase (pRL-TK) to normalize transfection. At 48 hpt, cells were lysed, and luciferase activities were measured by the Dual-Luciferase reporter assay (Figure 6A). The antagonistic effects of pCoV-N on pZAP were compared based on the relative fold of inhibition. The result showed that PEDV-N and TGEV-N can reduce the inhibition of luciferase signal by ZAP by about 25%, whereas SADS-CoV-N can reduce the relative inhibition up to 50% (Figure 6B). The antagonistic effects of these pCoV-Ns against pZAPL or pZAPS are comparable. Overall, the results showed the conserved antagonizing function of pCoV-Ns against both isoforms of pZAP.

### pCoV-Ns can alleviate zinc-finger antiviral protein-mediated suppression of influenza virus PB2 expression

**FIGURE 7:**
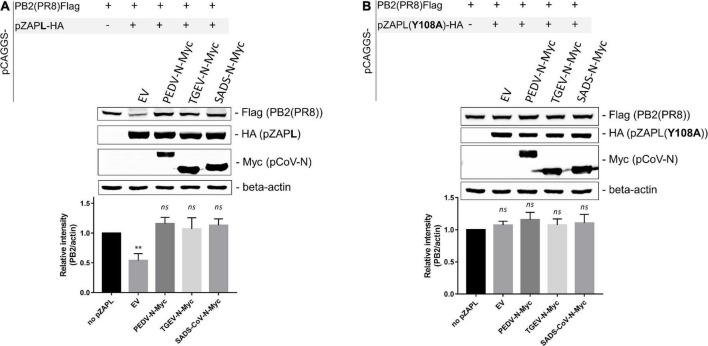
pCoV-Ns reverses the repression of PB2 expression by zinc-finger antiviral protein (ZAP). **(A,B)** A pCAGGS plasmid expressing Flag-tagged PB2 was co-transfected into HEK293T cells with a plasmid expressing HA-tagged pZAPL **(A)** or the non-functional ZAP mutant, ZAP (Y108A) **(B)**, and a plasmid encoding the indicated pCoV-N protein. An empty vector plasmid served as a control. At 48 hpt, cells were lysed, and protein expression was analyzed by SDS-PAGE/western blotting. The western blotting results shown are representative of three independent experiments. The relative band intensities of PB2-FLAG and beta-actin under each lane were analyzed using Image Lab software (Bio-Rad Laboratories, CA, United States). Values shown are the means ± SD of three independent measurements (Student’s *t*-test, **p < 0.005).

To demonstrate that pCoV-Ns can suppress the function of pZAP, we performed another assay based on the previous finding that influenza virus polybasic protein 2 (PB2) expression can be suppressed by ZAP via a viral mRNA reduction-dependent mechanism (Tang et al., 2017). HEK293T cells were co-transfected with a plasmid expressing Flag-tagged PB2 protein and the indicated pZAP variants in the absence or presence of a plasmid expressing various pCoV-N. The pCAGGS-empty vector was used as a mock control. SDS-PAGE/western blot analysis of the transfected cell lysates showed that PB2 expression was substantially reduced in the presence of pZAPL (Lane 2 in Figure 7A), consistent with the previous reports (Liu et al., 2015; Tang et al., 2017). However, in the presence of nucleocapsid proteins from PEDV, TGEV, or SADS-CoV, PB2 expression was restored to the same level as in the absence of ZAP (cf. Lanes 3-5 and 1, Figure 7A). To rule out the possibility that CoV-Ns contribute to enhancing PB2 expression independently of the ZAP-regulatory pathway, we replaced ZAP with a ZAP mutant [pZAPL(Y108A)] lacking the essential residue for RNA binding, rendering it non-functional (Luo et al., 2020; Figure 7B). The result showed that PB2 expression was not affected by the non-functional mutant [pZAPL(Y108A)] (Lane 2, Figure 7B) and that CoV-N only slightly increased the expression of PB2 compared to that with pZAPL (Lanes 3–5, Figure 7B). These results support the notion that the nucleocapsid proteins of porcine alpha-coronaviruses, at least of PEDV, TGEV, or SADS-CoV, have a conserved function in antagonizing the function of pZAP and are able to restore the expression of a ZAP-responsive protein such as PB2.

**FIGURE 8:**
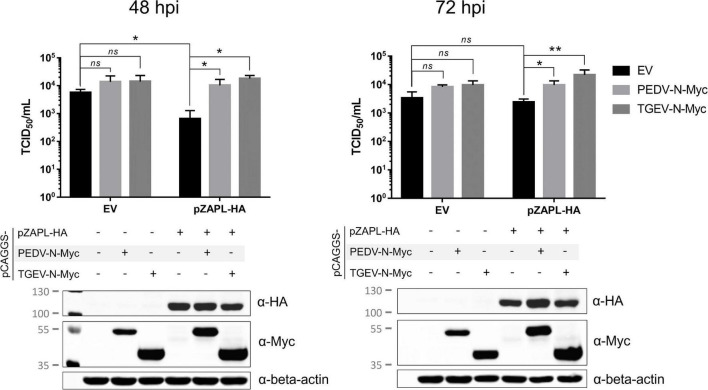
Suppression of CPD-porcine epidemic diarrhea virus (PEDV) growth by pZAPL can be reversed by pCoV-Ns. VeroE6 ZAP-KO cells were transfected with the indicated plasmids. At 24 hpt, transfected cells were infected with CPD-PEDV (MOI = 0.001). Supernatants were collected at 48 and 72 hpi to determine viral titers using the TCID_50_ assay. Expression of the indicated proteins is shown by SDS-PAGE/western blot results below. Values shown are averages ± SEM of three independent experiments (Student’s *t*-test, *p < 0.05, **p < 0.005).

### Growth suppression of CPD-porcine epidemic diarrhea virus by pZAPL can be reversed by pCoV-Ns

We next examined whether pCoV-Ns expressed *in-trans* can reverse the growth suppressive effect of ZAP on CPD-PEDV. VeroE6 ZAP-KO were co-transfected with HA-tagged pZAPL or empty vector and Myc-tagged PEDV-N or TGEV-N. At 24 hpt, transfected cells were inoculated with CPD-PEDV (MOI = 0.001). Supernatants containing viruses at 48 and 72 hpi were collected for viral titer analysis by TCID_50_. At 48 hpi, virus titers from cells expressing only pZAPL-HA were significantly lower than those from the mock-transfected cells indicating viral suppression by pZAPL (Figure 8). In the absence of pZAPL-HA expression, transfection of either PEDV-N-Myc or TGEV-N-Myc slightly but not statistically significantly increased viral titers. However, in the presence of pZAPL-HA, co-transfection with pCoV-Ns can significantly reverse the viral suppression induced by pZAPL-HA (Figure 8, left). Similar to the titers observed at 48 hpi, expression of pCoV-N without the suppressive effect of pZAPL-HA did not show significantly increased viral titers at 72 hpi. At 72 hpi, the effect of transient expression of pZAPL-HA did not significantly decrease viral titer compared with mock transfection, possibly because the virus produced sufficient N at this stage to target ZAP. However, both pCoV-Ns can still slightly increase viral titers in the presence of pZAPL compared with the condition where no additional pCoV-N was present. This result suggests that both PEDV-N and TGEV-N expressed *in-trans* may antagonize the function of pZAPL in suppressing the growth of CPD-PEDV.

## Discussion

The global threat posed by SARS-CoV-2 again underscores the need for a better understanding of coronavirus-host interaction. As an example, insights gained from previous SARS-CoV research can make a critical leap in the development of therapeutic and preventive approaches to alleviate the global suffering of COVID-19. A comprehensive understanding of how a host defends itself against viral infection and how viruses counteract this defense would better prepare us for the next pandemic.

Among various host antiviral factors, ZAP plays an important role in inhibiting the growth of many RNA viruses (Ficarelli et al., 2021), including SARS-CoV-2 (Nchioua et al., 2020), by suppressing the production of genomic or subgenomic viral RNA during viral infection. In this study, we demonstrated that the growth of PEDV can be suppressed by endogenous or porcine cell-derived ZAP in VeroE6 cells. The conserved function of ZAPs from both cell lines may be due to the conserved RNA-binding domain in the N-terminal part of ZAP in mammalian hosts (Gonçalves-Carneiro et al., 2021).

Two major isoforms of ZAP (short and long isoforms) contribute to viral restriction function. Despite sharing a common RNA-binding domain, the long isoform exerts stronger viral inhibition due to the extended C-terminal domain containing the inactive poly-(ADP-ribose) polymerase (PARP)-like domain, which has been shown to aid in RNA binding, interact with its cofactor (Kmiec et al., 2021) and recruit an endonuclease protein to degrade bound RNA (Ficarelli et al., 2019). Moreover, the long isoform is constitutively expressed, while the short isoform is dependent on IFN response (Ryman et al., 2005; Wang et al., 2010; Hayakawa et al., 2011; Schwerk et al., 2019). Considering that the VeroE6 cell line is IFN gene deficient (Emeny and Morgan, 1979; Chew et al., 2009), the long isoform, which is expressed at a much higher level (Figure 1A), would play a dominant role in virus inhibition in our experimental setup. Therefore, our study focused on the interplay between the virus and the long isoform of ZAP, with the IFN-dependent response playing a minor role. However, the contribution of pZAPS could not be excluded in a native host in which the IFN response functions normally.

Our analysis of the PEDV genomes revealed high CpG clusters in the 5′UTR, the ribosomal frameshift region and the structural gene region, particularly in the N gene. This pattern of CpG distribution is similar to that of the SARS-CoV-2 genome (Nchioua et al., 2020; Zimmer et al., 2021). This suggests that ZAP targets these regions of high CpG content and mediates the degradation of viral RNA, leading to suppression of viral growth, as found in SARS-CoV-2. We have shown that increasing CpG content only in the N gene can make viral RNA more visible to ZAP and thus more susceptible to degradation. Given that all coronaviral genomic and subgenomic RNA species share the N gene at the 3′-terminal segment, any change in the N gene could affect all species of viral RNAs. Therefore, a subtle effect of small CpG changes in the N gene could accumulate to cause sufficient suppression by ZAP, resulting in decreased replicative viral fitness of CPD-PEDV. This is supported by another study showing that SARS-CoV-2 containing a partial fragment with high CpG in its genome was attenuated in animal experiments (Trimpert et al., 2021). Therefore, the increased density of CpG in a specific genomic region rather than the CpG number in the whole genome could alter the ZAP susceptibility of PEDV. Our results have shown that the attenuation of CPD virus is mainly due to ZAP, as the suppressed viral replication can be reversed in ZAP-KO cells, but is evident in VeroE6 with exogenous pZAPL reconstitution. The attenuation shown here by designing CPD-PEDV could be applied based on the concept of *synthetic attenuated virus engineering* (SAVE) (Coleman et al., 2008) to generate an attenuated live vaccine candidate.

We have previously shown that PEDV-N or TGEV-N can promote PEDV replication by increasing the level of viral RNA in the viral replication process compared to the state without exogenous N-protein expression (Sungsuwan et al., 2020). Our findings from this work reveal an alternative factor that contributes to our previous observation that the additional CoV-N antagonizes ZAP and protects viral RNA from degradation. This novel function of pCoV-N has been shown to be conserved among porcine alpha-coronaviruses, including PEDV, TGEV, and SADS-CoV and likely among other human coronaviruses. We have also shown that the host expressing PEDV-N or TGEV-N *in-trans* can recover the slow replication of the CPD-virus. It could be argued that the extra PEDV-N supplements the delayed production of PEDV-N from the CPD-N gene and restores slow virus replication by participating in virion assembly. However, TGEV-N, which was previously shown to be unable to replace PEDV-N in the production of PEDV virions (Sungsuwan et al., 2020), also showed a similar effect. Together with the demonstration that these pCoV-Ns can restore normal expression of IAV-PB2, an unrelated protein known to be suppressed by ZAP, these results suggest a novel function of pCoV-Ns that antagonizes the pZAP function in a universal and non-pCoV-specific manner.

Our results showed that PEDV can grow faster in the ZAP-KO cells compared to the wild-type cells in the early phase of infection (24–48 hpi), while it reaches a plateau thereafter (Figures 1, 4). In the transient expression experiment, the suppression by ZAP is less significant in the late phase of virus replication (at 72 hpi, Figure 8). This may suggest that novel function of pCoV-N in antagonizing the function of ZAP may be an auxiliary role in addition to its primary functions of initiating or modulating viral genome replication (McBride et al., 2014). These primary functions of virus-derived N could be of high priority during the early phase of replication, when N production cannot catch up to antagonize the active ZAP in the host. However, once CoV-N produced either by the virus or by the host (*in-trans*) is excessive, ZAP inhibition may be observed. On the other hand, given that a primary function of CoV-N is to bind the viral RNA, it is also possible that the excess CoV-N may be sufficient to cover and protect the viral RNA from being recognized by ZAP as well.

Regarding the interaction between pZAPL and PEDV-N shown in this study, we could not exclude that it is direct or mediated by an intermediary. On the one hand, several works have shown that CoV-N proteins have myriads of interaction partners (McBride et al., 2014). On the other hand, two independent studies have shown that nucleocapsid proteins of coronaviruses (MERS- and SARS-CoVs) can interact with TRIM25, an essential ZAP co-factor (Li et al., 2017), preventing it from interacting with RIG-I for IFN production (Hu et al., 2017; Chang et al., 2020). This led us to hypothesize that PEDV-N might interact with pZAPL possibly via TRIM25 (Li et al., 2017; Zheng et al., 2017; Ficarelli et al., 2019). Whether pCoV-N interacts with TRIM25 and affects its cofactor function for ZAP requires further investigation.

In summary, PEDV can be suppressed by ZAP. Increasing CpG content only in the N gene makes the virus more susceptible to ZAP. Our study revealed an alternative function of the nucleocapsid protein (pCoV-N) that targets and counteracts the antiviral activity of ZAP. The insights into the interplay between coronavirus and host demonstrated in this work could be used to develop therapeutic and preventive agents to combat the impending pandemic.

## Data availability statement

The original contributions presented in this study are included in the article/Supplementary material, further inquiries can be directed to the corresponding author.

## Author contributions

SS: conceptualization, investigation, and methodology. SS and SK: validation. WM: bioinformatic analysis. SS and PJ-A: writing – original draft preparation. SS, PJ-A, and AJ: writing – review and editing. All authors have read and approved the manuscript.

## Footnotes

1
http://n2t.net/addgene:52961

2
http://citebay.com/how-to-cite/python/

## References

[B1] Chang C.-Y. Liu H. M. Chang M.-F. Chang S. C. (2020). Middle East Respiratory Syndrome Coronavirus Nucleocapsid Protein Suppresses Type I and Type III Interferon Induction by Targeting RIG-I Signaling. *J Virol.* 94 e00099–20. 10.1128/JVI.00099-20 32295922 PMC7307178

[B2] Chen S. Xu Y. Zhang K. Wang X. Sun J. Gao G. (2012). Structure of N-terminal domain of ZAP indicates how a zinc-finger protein recognizes complex RNA. *Struct. Mol. Biol.* 19 430–435. 10.1038/nsmb.2243 22407013

[B3] Cheng X. Virk N. Chen W. Ji S. Ji S. Sun Y. (2013). CpG usage in RNA viruses: Data and hypotheses. *PLoS One* 8:e74109. 10.1371/journal.pone.0074109 24086312 PMC3781069

[B4] Chew T. Noyce R. Collins S. E. Hancock M. H. Mossman K. L. (2009). Characterization of the interferon regulatory factor 3-mediated antiviral response in a cell line deficient for IFN production. *Mol. Immunol.* 46 393–399. 10.1016/j.molimm.2008.10.010 19038458

[B5] Coleman J. R. Papamichail D. Skiena S. Futcher B. Wimmer E. Mueller S. (2008). Virus Attenuation by Genome-Scale Changes in Codon Pair Bias. *Science* 320:1784. 10.1126/science.1155761 PMC2754401 18583614

[B6] Emeny J. M. Morgan M. J. (1979). Regulation of the Interferon System: Evidence that Vero Cells have a Genetic Defect in Interferon Production. *J. Gen. Virol.* 43 247–252. 10.1099/0022-1317-43-1-247 113494

[B7] Ficarelli M. Neil S. J. D. Swanson C. M. (2021). Targeted Restriction of Viral Gene Expression and Replication by the ZAP Antiviral System. *Annu. Rev. Virol.* 8 265–283. 34129371 10.1146/annurev-virology-091919-104213

[B8] Ficarelli M. Wilson H. Pedro Galao R. Mazzon M. Antzin-Anduetza I. Marsh M. (2019). KHNYN is essential for the zinc finger antiviral protein (ZAP) to restrict HIV-1 containing clustered CpG dinucleotides. *elife* 8:e46767. 10.7554/eLife.46767 31284899 PMC6615859

[B9] Gonçalves-Carneiro D. Takata M. A. Ong H. Shilton A. Bieniasz P. D. (2021). Origin and evolution of the zinc finger antiviral protein. *PLoS Pathogens* 17:e1009545. 10.1371/journal.ppat.1009545 33901262 PMC8102003

[B10] Guo X. Carroll J. W. Macdonald M. R. Goff S. P. Gao G. (2004). The zinc finger antiviral protein directly binds to specific viral mRNAs through the CCCH zinc finger motifs. *J. Virol.* 78 12781–12787. 15542630 10.1128/JVI.78.23.12781-12787.2004 PMC525010

[B11] Hayakawa S. Shiratori S. Yamato H. Kameyama T. Kitatsuji C. Kashigi F. (2011). ZAPS is a potent stimulator of signaling mediated by the RNA helicase RIG-I during antiviral responses. *Nat. Immunol.* 12 37–44. 10.1038/ni.1963 21102435

[B12] Hu Y. Li W. Gao T. Cui Y. Jin Y. Li P. (2017). The Severe Acute Respiratory Syndrome Coronavirus Nucleocapsid Inhibits Type I Interferon Production by Interfering with TRIM25-Mediated RIG-I Ubiquitination. *J. Virol.* 91 e2143–e2116. 10.1128/JVI.02143-16 PMC5375661 28148787

[B13] Jaru-Ampornpan P. Jengarn J. Wanitchang A. Jongkaewwattana A. (2017). Porcine Epidemic Diarrhea Virus 3C-Like Protease-Mediated Nucleocapsid Processing: Possible Link to Viral Cell Culture Adaptability. *J. Virol.* 91:2. 10.1128/JVI.01660-16 27807240 PMC5215342

[B14] Jengarn J. Wongthida P. Wanasen N. Frantz P. N. Wanitchang A. Jongkaewwattana A. (2015). Genetic manipulation of porcine epidemic diarrhoea virus recovered from a full-length infectious cDNA clone. *J. Gen. Virol.* 96 2206–2218. 10.1099/vir.0.000184 25979733

[B15] Kmiec D. Lista M. J. Ficarelli M. Swanson C. M. Neil S. J. D. (2021). S-farnesylation is essential for antiviral activity of the long ZAP isoform against RNA viruses with diverse replication strategies. *PLoS Pathogens* 17:e1009726. 10.1371/journal.ppat.1009726 34695163 PMC8568172

[B16] Li M. M. H. Lau Z. Cheung P. Aguilar E. G. Schneider W. M. Bozzacco L. (2017). TRIM25 Enhances the Antiviral Action of Zinc-Finger Antiviral Protein (ZAP). *PLoS Pathogens* 13:e1006145. 10.1371/journal.ppat.1006145 28060952 PMC5245905

[B17] Liu C.-H. Zhou L. Chen G. Krug R. M. (2015). Battle between influenza A virus and a newly identified antiviral activity of the PARP-containing ZAPL protein. *Proc. Natl. Acad. Sci. U.S.A.* 112:14048. 10.1073/pnas.1509745112 26504237 PMC4653199

[B18] Liwnaree B. Narkpuk J. Sungsuwan S. Jongkaewwattana A. Jaru-Ampornpan P. (2019). Growth enhancement of porcine epidemic diarrhea virus (PEDV) in Vero E6 cells expressing PEDV nucleocapsid protein. *PLoS One* 14:e0212632. 10.1371/journal.pone.0212632 30840701 PMC6402621

[B19] Luo X. Wang X. Gao Y. Zhu J. Liu S. Gao G. (2020). Molecular Mechanism of RNA Recognition by Zinc-Finger Antiviral Protein. *Cell Rep.* 30 46–52.e4. 31914396 10.1016/j.celrep.2019.11.116

[B20] McBride R. van Zyl M. Fielding B. (2014). The Coronavirus Nucleocapsid Is a Multifunctional Protein. *Viruses* 6:2991. 10.3390/v6082991 PMC4147684 25105276

[B21] Moura G. Pinheiro M. Arrais J. Gomes A. C. Carreto L. Freitas A. (2007). Large Scale Comparative Codon-Pair Context Analysis Unveils General Rules that Fine-Tune Evolution of mRNA Primary Structure. *PLoS One* 2:e847. 10.1371/journal.pone.0000847 17786218 PMC1952141

[B22] Nchioua R. Kmiec D. Müller J. A. Conzelmann C. Groß R. Swanson C. M. (2020). SARS-CoV-2 Is Restricted by Zinc Finger Antiviral Protein despite Preadaptation to the Low-CpG Environment in Humans. *mBio* 11 e1930–e1920. 10.1128/mBio.01930-20 33067384 PMC7569149

[B23] Reed L. J. Muench H. A. (1938). A SIMPLE METHOD OF ESTIMATING FIFTY PER CENT ENDPOINTS. *Am. J. Epidemiol.* 27 493–497. 10.1016/j.jviromet.2005.05.005 15955576

[B24] Rima B. K. McFerran N. V. (1997). Dinucleotide and stop codon frequencies in single-stranded RNA viruses. *J. Gen. Virol.* 78 2859–2870. 10.1099/0022-1317-78-11-2859 9367373

[B25] Ryman K. D. Meier K. C. Nangle E. M. Ragsdale S. L. Korneeva N. L. Rhoads R. E. (2005). Sindbis virus translation is inhibited by a PKR/RNase L-independent effector induced by alpha/beta interferon priming of dendritic cells. *J. Virol.* 79 1487–1499. 10.1128/JVI.79.3.1487-1499.2005 15650175 PMC544143

[B26] Schwerk J. Soveg F. W. Ryan A. P. Thomas K. R. Hatfield L. D. Ozarkar S. (2019). RNA-binding protein isoforms ZAP-S and ZAP-L have distinct antiviral and immune resolution functions. *Nat. Immunol.* 20 1610–1620. 10.1038/s41590-019-0527-6 31740798 PMC7240801

[B27] Simmonds P. (2012). SSE: A nucleotide and amino acid sequence analysis platform. *BMC Res. Notes* 5:50. 10.1186/1756-0500-5-50 22264264 PMC3292810

[B28] Sungsuwan S. Jongkaewwattana A. Jaru-Ampornpan P. (2020). Nucleocapsid proteins from other swine enteric coronaviruses differentially modulate PEDV replication. *Virology* 540 45–56. 10.1016/j.virol.2019.11.007 31756532 PMC7112109

[B29] Takata M. A. Goncalves-Carneiro D. Zang T. M. Soll S. J. York A. Blanco-Melo D. (2017). CG dinucleotide suppression enables antiviral defence targeting non-self RNA. *Nature* 550 124–127. 10.1038/nature24039 28953888 PMC6592701

[B30] Tang Q. Wang X. Gao G. (2017). The Short Form of the Zinc Finger Antiviral Protein Inhibits Influenza A Virus Protein Expression and Is Antagonized by the Virus-Encoded NS1. *J. Virol.* 91 e01909–16. 10.1128/JVI.01909-16 27807230 PMC5215320

[B31] Trimpert J. Dietert K. Firsching T. C. Ebert N. Thi Nhu Thao T. Vladimirova D. (2021). Development of safe and highly protective live-attenuated SARS-CoV-2 vaccine candidates by genome recoding. *Cell Rep.* 36:109493. 10.1016/j.celrep.2021.109493 34320400 PMC8289629

[B32] Wang N. Dong Q. Li J. Jangra R. K. Fan M. Brasier A. R. (2010). Viral induction of the zinc finger antiviral protein is IRF3-dependent but NF-kappaB-independent. *J. Biol. Chem.* 285 6080–6090. 10.1074/jbc.M109.054486 20048147 PMC2825402

[B33] Xia X. (2020). Extreme Genomic CpG Deficiency in SARS-CoV-2 and Evasion of Host Antiviral Defense. *Mol. Biol. Evol.* 37 2699–2705. 32289821 10.1093/molbev/msaa094 PMC7184484

[B34] Xie L. Lu B. Zheng Z. Miao Y. Liu Y. Zhang Y. (2018). The 3C protease of enterovirus A71 counteracts the activity of host zinc-finger antiviral protein (ZAP). *J. Gen. Virol.* 99 73–85. 10.1099/jgv.0.000982 29182509

[B35] Zhao Y. Song Z. Bai J. Liu X. Nauwynck H. Jiang P. (2020). Porcine reproductive and respiratory syndrome virus Nsp4 cleaves ZAP to antagonize its antiviral activity. *Vet. Microbiol.* 250:108863. 10.1016/j.vetmic.2020.108863 33035816

[B36] Zheng X. Sun Z. Yu L. Shi D. Zhu M. Yao H. (2021). Interactome Analysis of the Nucleocapsid Protein of SARS-CoV-2 Virus. *Pathogens* 10:1155. 10.3390/pathogens10091155 PMC8465953 34578187

[B37] Zheng X. Wang X. Tu F. Wang Q. Fan Z. Gao G. (2017). TRIM25 Is Required for the Antiviral Activity of Zinc Finger Antiviral Protein. *J. Virol.* 91 e88–e17. 10.1128/JVI.00088-17 PMC5391446 28202764

[B38] Zimmer M. M. Kibe A. Rand U. Pekarek L. Ye L. Buck S. (2021). The short isoform of the host antiviral protein ZAP acts as an inhibitor of SARS-CoV-2 programmed ribosomal frameshifting. *Nat. Commun.* 12:7193. 10.1038/s41467-021-27431-0 34893599 PMC8664833

