# Supplementary material for: Zinc-finger antiviral protein-mediated inhibition of porcine epidemic diarrhea virus growth is antagonized by the coronaviral nucleocapsid protein
Source: Front Microbiol. 2022 Sep 8;13:975632. doi: 10.3389/fmicb.2022.975632 (PMC9493364; doi:10.3389/fmicb.2022.975632)
Supplement: Supplementary file 1 [file Table_1.DOCX]

Supplementary Material

**Table S1.** Sequences of all primers used.

| **Primer name** | **Sequence** |
| --- | --- |
| pZAP-F | TGTCTCATCAACGCGACCATGGCTGACCCGGGGG |
| pZAPL-R | ACGTCGTAGGGGTAGGTACCACTGATCACGCAGGGTTTGTC |
| pZAPS-R | ACGTCGTAGGGGTAGGTACCGCCCATTCCCCGTTTCATCTG |

**
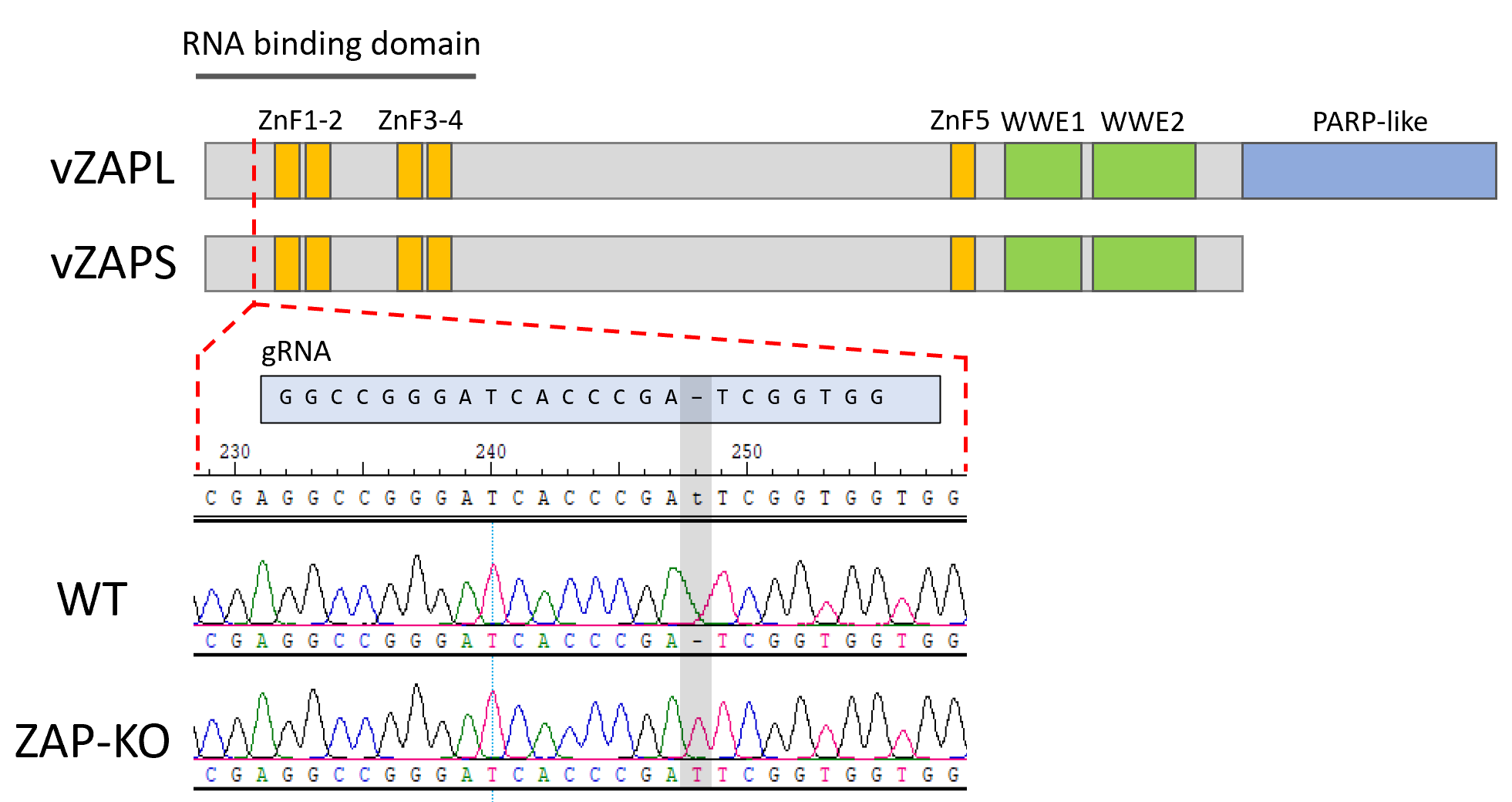
**

**Supplementary Figure S1.** Sequencing analysis of cellular RNA from ZAP gene derived from the ZAP-KO VeroE6 cell line, generated by CRISPR-Cas9 based technique. A clone of ZAP-KO VeroE6 cell line verified by western-blot analysis was harvested for cellular RNA extraction. The extracted cellular RNA was reverse-transcribed to generated DNA fragment covering targeted region by sgRNA. The sequence of DNA fragment was analyzed by Sanger sequencing.


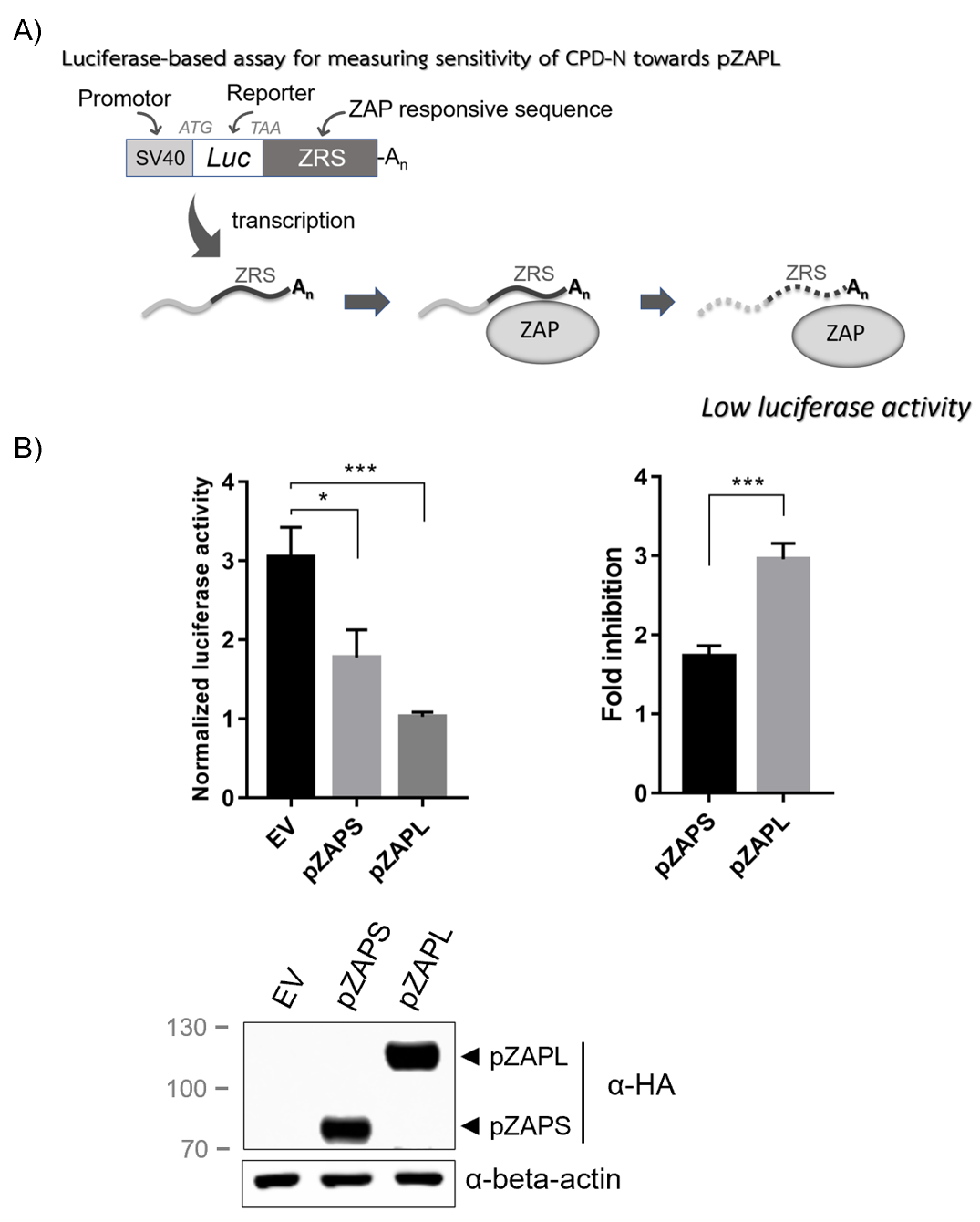


**Supplementary Figure S2.** A) Schematic picture showing a luciferase-based assay for verifying activities of pZAPL and -S. ZRS in the RNA, encoded from pGL3-Luc-ZRS plasmid, will be targeted by an active ZAP for entire-RNA degradation, resulting in lower luciferase activity. B) pGL3-luc-ZRS (ZRS = N-0.10 (Figure 4C)) was co-transfected with a transfection-normalizing plasmid, pRL-TK, and empty control plasmid (EV) or pCAGGS-pZAPL-HA or pCAGGS-pZAPS-HA into ZAP-KO-HEK293T cells. At 48 hpt, cells were lysed and measured for luciferase activity by a Dual-luciferase reporter assay. Sensitivity of the ZRS towards pZAPS/L was compared as inhibition fold, calculated as described in Methods. Fold inhibition are means ± SD of three independent measurements (Student’s t-test, **p* < 0.05, ***p* < 0.005, ****p* < 0.0005). (*bottom*) SDS-PAGE/Western blot showing expression of pZAPS/L.

**CPD-PEDV-N sequences**

**N-0.05** ATGGCTTCTGTCAGCTTTCAGGATCGTGGCCGCAAACGCGTGCCATTATCTCTCTATGCGCCACTTAGGGTTACTAATGACAAGCCCCTTTCTAAGGTACTTGCAAACAACGCTGTACCGACTAACAAGGGGAATAAGGACCAGCAAATTGGGTACTGGAATGAGCAAATTCGCTGGCGCATGCGCCGTGGTGAGCGAATTGAACAACCGTCCAATTGGCATTTCTACTACCTCGGAACAGGACCTCACGGCGACCTCCGTTATAGGACTCGGACTGAGGGTGTTTTCTGGGTTGCTAAAGAAGGCGCTAAGACTGAACCCACTAATTTGGGTGTCAGAAAGGCGTCTGAAAAGCCAATCATACCAAAATTCTCTCAACAGCTCCCCATTGTGGTTGAGATTGTGGAACCTAACACACCTCCTGCTTCACGTGCAAATTCGCGTAGCAGGAGTCGTGGCAATGGCAACAATAGGTCTCGGTCTCCAAGTAACAACAGAGGCAATAACCAGTCCCGTGGTAATTCACAGAATCGTGGAAATAACCAGGGTCGTGGCGCTTCTCAGAACAGAGGAGGCAATAATAATAACAATAACAAGTCTCGTAACCAGTCCAATAACAGGAACCAGTCAAATGACCGTGGTGGTGTAACATCACGCGATGATCTGGTGGCGGCTGTCAAGGATGCACTTAAATCTTTGGGTATTGGAGAAAATCCTGACAGGCATAAGCAACAGCAGAAGCCTAAGCAGGAAAAGTCTGACAACAGCGGCAAAAATACACCTAAGAAGAATAAATCCAGGGCCACTTCGAAGGAACGTGACCTCAAAGACATCCCAGAGTGGAGGAGAATTCCCAAGGGCGAAAATAGCGTAGCAGCTTGCTTCGGACCCAGAGGGGGCTTCAAAAACTTTGGTGATGCGGAATTTGTCGAAAAAGGGGTTGATGCGTCAGGCTATGCTCAGATCGCCAGTTTAGCGCCAAATGTTGCAGCATTGCTCTTTGGTGGTAACGTGGCTGTTCGTGAGCTGGCGGATTCTTACGAAATTACATACAACTATAAAATGACTGTGCCAAAGTCAGATCCGAATGTCGAGCTTCTTGTTTCACAGGTGGATGCGTTTAAAACTGGGAATGCAAAACTCCAGAGAAAAAAGGAAAAGAAAAACAAGCGTGAAACCACGCTGCAGCAGCATGAAGAGGCGATCTACGATGATGTGGGTGCGCCATCTGATGTGACCCATGCCAATCTGGAATGGGACACAGCTGTTGACGGTGGTGATACGGCCGTTGAAATTATCAACGAGATCTTCGATACAGGAAATTAA

**N-0.10**

ATGGCTTCTGTGAGCTTTCAGGATCGTGGCCGCAAACGCGTGCCACTGTCTCTGTATGCCCCTCTTCGCGTTACTAATGACAAGCCGCTTTCTAAGGTACTTGCAAACAACGCTGTACCCACTAACAAGGGGAATAAGGACCAGCAAATAGGGTACTGGAATGAGCAAATTCGCTGGCGGATGCGCCGTGGTGAGCGAATTGAACAACCTTCGAATTGGCATTTCTACTACCTCGGAACAGGACCTCACGGCGATCTCCGTTATAGGACTCGGACTGAGGGTGTGTTCTGGGTCGCGAAAGAGGGCGCTAAGACTGAACCCACTAATCTGGGTGTGCGTAAGGCGTCTGAAAAGCCAATCATACCAAAATTCTCTCAACAGCTCCCGATTGTAGTTGAGATTGTTGAACCTAACACACCTCCTGCTTCACGGGCAAATTCGCGTAGCAGGAGTCGTGGCAATGGCAACAATAGGTCTAGATCTCCATCGAACAATCGGGGTAATAACCAGTCCCGTGGTAATTCACAGAATCGTGGAAATAACCAGGGTCGTGGAGCTTCTCAGAACAGAGGAGGCAATAATAATAACAATAACAAGTCTCGTAACCAATCGAATAACCGGAACCAGTCAAATGACCGGGGTGGTGTAACATCACGCGATGATCTGGTGGCTGCGGTCAAGGATGCACTTAAATCTCTCGGTATTGGAGAAAATCCTGACAGGCATAAGCAACAGCAGAAGCCTAAGCAGGAAAAAAGCGACAATAGCGGCAAAAATACACCTAAGAAGAACAAATCCAGGGCCACTAGTAAGGAACGTGACCTCAAAGACATCCCCGAGTGGAGGCGGATACCCAAGGGCGAAAATAGCGTCGCAGCTTGCTTCGGACCCAGGGGGGGCTTCAAAAACTTTGGAGATGCGGAATTTGTCGAAAAAGGTGTTGATGCGTCAGGCTATGCTCAAATCGCCAGTCTGGCACCGAATGTTGCAGCATTGCTGTTTGGTGGTAATGTGGCTGTTCGTGAGCTGGCTGACTCTTACGAGATTACATATAACTATAAGATGACTGTGCCAAAGTCAGATCCAAATGTTGAGCTTCTGGTTTCACAGGTGGACGCTTTTAAAACTGGGAACGCAAAACTCCAGAGAAAAAAGGAAAAGAAAAACAAGCGTGAAACCACGCTGCAGCAGCATGAAGAGGCCATCTACGACGACGTCGGTGCGCCATCTGATGTGACCCACGCCAATCTGGAATGGGACACAGCTGTTGATGGTGGTGATACCGCCGTCGAAATTATCAACGAGATCTTCGATACAGGAAATTAA
